# Implementation of African Swine Fever Biosecurity and Post-Outbreak Recovery in Vietnam, Malaysia, and the Philippines

**DOI:** 10.3390/vetsci13101113

**Published:** 2026-10-18

**Authors:** Keyi Duan, Ting Lin, Zengyong Zhu, Karma Rinzin, Ashish Sutar, Waroonsiri Charoenlarp

**Affiliations:** 1 Institute of Animal Science, Chinese Academy of Agricultural Sciences (CAAS), Beijing 100193, China; 2 College of Economics and Management, China Agricultural University, Beijing 100083, China; linting20210301@163.com; 3 World Organisation for Animal Health, Sub-Regional Representation for South-East Asia, Bangkok 10400, Thailand

**Keywords:** African swine fever, biosecurity, pig production, farm recovery, restocking, policy

## Abstract

African swine fever (ASF) continues to disrupt pig production in Southeast Asia. Control measures and conditions for recovery vary across production systems. This qualitative multiple-case study examined 24 purposively selected production units in Vietnam (n = 8), Malaysia (n = 12), and the Philippines (n = 4). Government and partner institutions facilitated access to the units. Face-to-face interviews and field notes collected between August 2025 and February 2026 were analysed alongside policy documents. National statistics described changes in pig inventories and output. In Vietnam, live-weight pig output in 2024 exceeded the 2018 level, although the national herd remained smaller. Recovery varied among the sampled units, with greater structural investment in integrated enterprises than in family farms. In Malaysia, nine sampled farms were operating, several were undergoing substantial upgrades, and three remained depopulated while awaiting approval to restock. In the Philippines, one commercial unit reported coordinated control measures and household measures. The three programme-linked backyard units relied on household measures and project support. Gaps in transport, reporting, disposal, and restocking arrangements limited the reach of farm-level measures across the cases.

## Simple Summary

African swine fever continues to affect pig farming in Southeast Asia, and farmers face different difficulties in controlling the disease and restarting production. This study examined 24 pig-production units in Vietnam, Malaysia, and the Philippines through interviews, farm visits, and policy documents. Larger farms reported investment in housing, entry controls, and transport, while family and backyard farms relied more on daily practices and available project support. Three Malaysian farms remained empty while awaiting permission to restock. Problems with animal transport, disease reporting, disposal of dead pigs, and restocking procedures continued to limit recovery. The findings suggest that farm improvements need support from veterinary services, clear reporting and compensation procedures, and practical restocking guidance.

## 1. Introduction

African swine fever (ASF), caused by African swine fever virus (ASFV), affects domestic pigs and wild boar, with case fatality rates that can approach 100% [1]. Deaths, compulsory culling, movement restrictions, and delayed restocking can reduce pork supply and disrupt trade [2]. Following its emergence in Asia in 2018, ASF spread to Southeast Asia in 2019 and has continued to affect pig production across the region [3,4]. World Animal Health Information System (WAHIS) records show continuing outbreaks in domestic and wild pigs [5]. FAOSTAT figures indicate that Southeast Asia held approximately 65.9 million pigs and produced 6.99 million tonnes of pig meat in 2024. Vietnam and the Philippines accounted for 53.8% and 19.3% of regional pig meat production, respectively [6]. Pig production in Southeast Asia includes integrated enterprises, independent commercial farms, family farms, and backyard holdings [4,7]. These producers differ in their facilities, financial resources, access to veterinary services, and links with traders and slaughterhouses. Smallholders remain important in several countries, including the Philippines, where they held 78.3% of the national pig inventory in 2025 [8]. International and regional ASF strategies cover surveillance, movement control, biosecurity, and outbreak response [9,10]. Their implementation requires investment and daily changes in farm management, together with veterinary services, reporting arrangements, and access to restocking approval.

Previous studies have reviewed have identified farm structure, animal movement, wildlife contact, surveillance, and biosecurity as factors affecting ASF introduction and spread [11,12]. Previous studies have examined the spatial and temporal patterns of ASF outbreaks in Vietnam and the Philippines [13,14]. Other research has assessed the economic effects of ASF and related policy responses in China [2], as well as the potential impacts of outbreaks on pork markets in Canada and the United States [15,16]. Behavioural research has shown how financial constraints, access to veterinary services, market pressure, and uncertainty influence prevention [17,18]. Further farm-level evidence is needed on how these factors interact during recovery. In particular, the links between changes in farm practices, institutional support, and the restoration of production require closer examination across different production systems. Producers may invest in housing and disinfection while continuing to face difficulties with transport, disease reporting, carcass disposal, or restocking. Understanding these difficulties can help identify where technical guidance, financial support, or administrative procedures need improvement.

Producer decisions are also shaped by perceptions of disease risk and the expected consequences of control measures. Reporting suspected ASF can lead to culling and business interruption, making compensation and restocking arrangements relevant to reporting decisions. This study used Protection Motivation Theory (PMT) to examine perceived disease threats and producers’ ability to respond [19,20], and the Theory of Planned Behaviour (TPB) to interpret attitudes, social norms, and perceived control over reporting and disposal [21]. Concepts of externalities and incentive compatibility helped explain the incentives associated with culling, compensation, and resuming production [22,23].

This qualitative study examined 24 purposively selected pig-production units in Vietnam, Malaysia, and the Philippines. The cases covered different production systems, reported ASF experiences, and recovery conditions. Interviews, field notes, and policy documents were used to examine practices across eight biosecurity domains, reported changes in investment and support, and the status of production recovery. The analysis focused on which measures were implemented, how producers obtained the resources and support needed to maintain or resume production, and which farm-level and institutional problems remained unresolved. The findings can inform support for producers managing ASF risks and rebuilding production under different local conditions.

## 2. Materials and Methods

### 2.1. Study Settings and Data Sources

This study examined pig production under different ASF control and recovery conditions in Vietnam, Malaysia, and the Philippines. Vietnam has both integrated enterprises and family farms [7,24]. In the Philippines, production is geographically dispersed, and smallholders account for most of the national herd [8,25]. Malaysia has a smaller pig industry [26]. The sampled Malaysian farms varied in size, investment, and restocking status. The study used official statistics, policy documents, interviews, and field notes. World Animal Health Information System (WAHIS) records provided regional outbreak information [5]. National production data included pig inventory and output figures for 2018 and 2024 from the National Statistics Office of Vietnam [24], swine inventory and pork output figures for 2020 and 2024 from the Department of Statistics Malaysia [26], and pig inventories for 1 January 2020 and 31 December 2025 from the Philippine Statistics Authority [8,25].

Policy sources included global and regional ASF strategies [9,10], Vietnam’s national ASF plan [27], and Malaysia’s veterinary protocol [28]. Malaysia’s annual veterinary report provided information on sector development [29]. Philippine administrative orders covered surveillance and compensation [30,31]. Programme materials described the CABI initiative [32] and ASF vaccine deployment in Vietnam [33]. This study included documents published or in effect through August 2026 that directly addressed surveillance, notification, movement, culling and disposal, compensation, vaccination, biosecurity, or restocking in the three countries. Interviews and field notes collected from August 2025 to February 2026 provided the original case evidence. Information published after fieldwork was used only to describe the statistical, outbreak, or policy context. For individual units, “after ASF” refers to a respondent-reported farm episode or restocking period when its timing was available. Otherwise, this study described practices observed or reported during the 2025–2026 visit without specifying when they were adopted. Pre-outbreak herd sizes were recorded where respondents provided them.

### 2.2. Case Selection and Interview Procedures

This study used a qualitative multiple-case design, with the pig-production unit as the unit of analysis. Purposive selection focused on production settings and experiences relevant to ASF prevention, control, and recovery [34]. Selection within each country reflected its production systems and the conditions affecting continued operation or restocking. Local veterinary and partner institutions assisted with farm contacts and fieldwork arrangements.

In Vietnam, selection considered geographical area, production type, scale, and reported recovery experience. The eight units included five integrated or commercial enterprises and three family farms. They provided information on farm management, investment, and recovery under different production arrangements.

In Malaysia, fieldwork covered Selangor, Penang, and Perak, which had substantial pig populations [35]. The selected farms in these states were operating during fieldwork. The nine operating units comprised six commercial farms and three family farms. Three depopulated farms in Melaka were also included to examine the measures taken after ASF, conditions for restocking, and the status of farms awaiting approval. The Malaysian sample therefore included 12 units.

In the Philippines, selection focused on backyard production because of its importance in the national pig sector [8,25]. Three backyard households in the Dumaguete area participated in the Community ASF Biosecurity Interventions (CABI) programme. Provincial statistics recorded 275,853 pigs in Negros Oriental on 1 January 2024, including 263,238 in smallholder farms [36]. The three units were included to examine household practices and community-based support. CABI is an FAO initiative launched in 2022 to develop practical biosecurity measures with smallholders and local animal-health partners [32]. The sample also included a large family-operated commercial farrow-to-finish farm in Batangas, which had reported ASF outbreaks in 2020 and 2023. This farm provided information on control and recovery in a larger production unit.

The sample comprised 8 units in Vietnam, 12 in Malaysia, and 4 in the Philippines. These country samples differed in size and production-system composition. The same analytical domains were used to organise evidence from all units, and findings were interpreted in relation to each unit’s production system, ASF history, and recovery status. Units were assigned codes VN1–VN8, MY1–MY12, and PH1–PH4 (Table 1). MY10–MY12 were included in the recovery analysis; current biosecurity practices were assessed only at the 21 operating units. The first and corresponding authors jointly conducted 24 face-to-face production-unit interviews between August 2025 and February 2026 using the structured guide in Supplementary File S1. Respondents received information about the study and gave verbal consent to participate and have the interviews recorded. Interviews were conducted in English without translation. The first author transcribed the recordings and supplemented them with notes taken during or immediately after each visit. Additional interviews with veterinary authorities clarified policy implementation. Philippine institutional informants also included representatives of farmer organisations and universities. Their number was not systematically recorded, and these interviews were excluded from the count of 24 production-unit interviews. The interview guide covered production, ASF experience, prevention, investment, and policy support. Information on some biosecurity topics was missing for individual units. These topics were recorded as undocumented; missing information was not taken to indicate that a practice was absent.

### 2.3. Analytical Framework

Information from interview transcripts, field notes, and policy documents was organised by case into eight analytical domains: (1) perimeter and zoning; (2) personnel entry; (3) vehicles and incoming materials; (4) pig movement and restocking; (5) feed and swill; (6) cleaning, disinfection, and pest control; (7) carcass, manure, wastewater, and environmental management; and (8) surveillance, reporting, training, and institutional support. These domains covered ASF transmission through direct contact, ingestion, contaminated objects, and environmental contamination, as well as surveillance and outbreak response [11,37]. The matrix recorded practices, potential risks, information sources, and dates of adoption where reported (Table 2).

The analysis examined documented practices and risks within each domain, including differences in implementation among units (Table 3). Interview coverage varied across domains and subtopics. Practices that were not mentioned in interviews or documented in other case materials were recorded as undocumented. Where a unit reported both protective and potentially risky practices within a domain, both were retained in the case description.

Production recovery was described for each unit using operating or depopulated status, restocking approval, and reported investment. Current herd size was compared with the respondent-reported pre-outbreak herd size where both figures were available.

**Table 2:** **Analytical domains and evidence** **recorded for each case.**

Domain	Evidence Considered	Transmission or Response Link
1. Perimeter and zoning	Barriers, production/residential separation, and zoning	Reduces direct contact with neighbouring, feral, or wild pigs and uncontrolled boundary crossings
2. Personnel entry	Visitor limits, showering, changing, and entry isolation	Reduces human-mediated transfer on hands, footwear, clothing, and personal equipment
3. Vehicles and materials	Vehicle ownership, route separation, cleaning, and material entry	Reduces mechanical transfer of infectious material on vehicles, crates, equipment, and incoming materials
4. Pig movement and restocking	Pig sourcing, loading, quarantine, movement, and re-entry approval	Reduces introduction and onward spread through infected pigs, loading, transport, and premature re-entry
5. Feed and swill	Feed sources, food waste, swill use, and treatment	Reduces oral exposure to contaminated pork, offal, swill, feed, or water
6. Cleaning, disinfection, and pest control	Cleaning frequency, product application, and pest exclusion	Removes organic contamination and inactivates virus on surfaces; pest exclusion may reduce mechanical carriage
7. Carcass and environmental management	Sick/dead-pig handling, carcass disposal, manure, and wastewater	Prevents exposure to infectious carcasses, body fluids, manure, wastewater, and contaminated sites
8. Surveillance, reporting, training, and support	Monitoring, notification, training, compensation, and support	Shortens time to detection, isolation, movement standstill, trace-back, and coordinated response

Notes: The examples identify topics sought in the interviews and field notes, not a checklist of measures verified at every unit. A missing account does not establish non-adoption; neither a reported practice nor an observed facility establishes consistent or effective implementation.

## 3. Results

### 3.1. National Production Context and Recovery Among Sampled Units

National production figures covered different reference years and indicators, with no common pre-outbreak baseline for ranking recovery or policy effectiveness. In Vietnam, pig inventory fell from 28.2 million head in 2018 to 26.5 million in 2024, a decrease of about 6%. Live-weight pig output increased from approximately 3.8 to 5.18 million tonnes over the same period, an increase of about 36% [24]. In Malaysia, pig inventory declined from approximately 1.87 million in 2020 to 1.16 million in 2024, and pork output fell from 220.6 to 136.5 thousand tonnes [26]. In the Philippines, pig inventory stood at 8.79 million head on 31 December 2025, compared with 12.80 million on 1 January 2020. Smallholders accounted for 78.3% of the 2025 herd [8,25].

Recovery was assessed for individual units. Vietnamese integrated enterprises reported controls requiring greater capital investment. Herd sizes at VN5 and VN6 were approximately 55% and below 30% of their respective earlier levels reported by respondents. Family farms mainly reported lower-cost routine measures. In Malaysia, nine sampled farms were operating, several following structural upgrades. MY10–MY12 remained depopulated while awaiting restocking approval. In the Philippines, the commercial farm reported coordinated controls, and the three CABI-linked backyard units used household measures supported by the project.

### 3.2. Documented Biosecurity Practices

Table 3 presents the main practices, risks, and information gaps recorded for the 21 operating units, organised by transmission or response pathway. It provides qualitative descriptions of individual cases. Cleaning and disinfection were widely discussed. Some interviews included details on feed transport or packaging, but information on feed sources or swill use was documented only for PH2 and PH3. These topics were insufficiently covered in the other 19 operating units. Coverage of pig movement and restocking, carcass and environmental management, and institutional support also varied among interviews. Missing information was recorded as undocumented.

MY10–MY12 were included in the recovery analysis. Current biosecurity implementation was not assessed at these depopulated units. Where timing was available, the analysis identified measures reported to have been introduced after a farm ASF episode. For many measures, adoption dates were unavailable, and only the practices in place at the fieldwork visit could be described.

**Table 3:** **Qualitative synthesis of documented** **biosecurity practices and evidence gaps among operating units.**

Domain	Illustrative Documented Practices	Illustrative Risks or Gaps	Information Limit
Perimeter and zoning	VN1 separated home and production areas; VN4 used staged entry zones. PH1 reported closed access.	VN2 lacked residential/production separation; damaged netting was observed at VN7–VN8.	Physical barriers were described more fully than their routine use; no comparable audit across units.
Personnel entry	MY1–MY2 reported three-day worker isolation, showering, and dedicated clothing; PH1 restricted entry.	VN2 reported no isolation procedure for people entering or leaving the farm.	Visitor and worker procedures were not equally documented at all units.
Vehicles and materials	VN4 used dedicated vehicles and loading areas; VN5 used external handover; MY1–MY2 used off-farm transfer.	VN2 mixed feed and pig transport; VN3 had crossed routes; VN7–VN8 mainly sprayed tyres.	No product concentration, contact time, or virological effectiveness was verified.
Pig movement and restocking	VN5 reported handover points outside the farm for breeding and fattening pigs.	Traders entered VN2; PH4 reported sales through traders or direct sale.	Sourcing, quarantine, and restocking details were incompletely recorded.
Feed and swill	PH2 reported no swill use; PH2–PH3 described locally grown forage.	VN2 used bagged feed and VN3 reused feed bags, but neither account establishes feed-source safety.	Substantive feed-source or swill-use practices were documented for only PH2–PH3 among 21 operating units; no adoption comparison is justified.
Cleaning, disinfection, and pest control	All operating Vietnamese and Malaysian units reported cleaning or disinfection; PH1 reported twice-daily application.	At VN7–VN8, damaged netting left potential pest entry; tyre-only spraying cannot establish whole-vehicle disinfection.	Frequency and scope were self-reported; products, organic-soil removal, and contact times were not audited.
Carcass and environmental management	A Malaysian farm described wastewater upgrades; a family-operated unit among MY6–MY9 reported burial away from the main farm area.	PH4 described private slaughter and distribution of meat from sick pigs; a Vietnamese respondent described low-price sale of suspected pigs.	Official disposal records and systematic manure/wastewater data were unavailable.
Surveillance, reporting, training, and support	VN6 and PH2–PH4 reported formal or project-linked training; PH1 reported government-supported vaccination.	Some respondents described delayed/non-notification or informal handling after suspected disease.	Programme scope, reporting records, compensation receipt, and vaccine details could not be independently verified.

Notes: Farm codes identify illustrative cases, not all units with a practice. Examples derive from respondent accounts and field notes unless stated otherwise; they were not independently audited. “Not documented” means that the study record is insufficient, not that a measure was absent. MY10–MY12 were depopulated and are excluded from current-practice synthesis. Timing is labelled only where the respondent or field notes supplied it; otherwise the table refers to reported or observed practice at fieldwork.

### 3.3. Farm-Level Biosecurity Measures in Vietnam

#### 3.3.1. Farm Housing, Zoning, and Personnel Access

Farm layout and entry procedures varied across the Vietnamese units. After its first reported ASF outbreak, VN1 separated residential and production areas and restricted access to household members. Anyone returning after leaving the premises was required to wash, disinfect, and remain isolated for one day before resuming farm work.

VN2 operated a farrow-to-finish system using traditional pig housing with hollow-floor pens, water-flushed manure handling, and water spraying for cooling. Its residential and production areas were not physically separated, and no isolation procedure was used for persons entering or leaving the farm. Pig traders entered the premises when pigs were collected for sale. VN3 had a relatively simple production layout: internal roads were unpaved, the residential and pig-production areas occupied the same farm space, and routes used by workers and vehicles crossed within the premises.

The commercial and integrated units reported several forms of physical and procedural separation. VN4 had installed shower and changing facilities, material drying rooms, automated vehicle cleaning and disinfection equipment, and a five-stage entry and isolation system. Personnel were required to shower and change clothing before entering the production area. The unit also used designated vehicles and separate loading and unloading areas. VN7 and VN8 used three-zone separation systems, Danish-style changing rooms, rodent-proof perimeter structures, and protective netting. During the field visits, sections of the protective netting were damaged, and some areas were not fully covered by video monitoring. VN5 had modified its personnel-entry arrangements over time. Under its earlier procedure, workers isolated for seven days before entering the farm and subsequently remained on the premises for three weeks. At the time of fieldwork, workers were permitted to return home daily but were required to disinfect before re-entering the farm.

#### 3.3.2. Cleaning, Disinfection, Pest Control, and Environmental Management

All eight Vietnamese units reported routine or intensified disinfection during the study period, although frequency and scope differed.

VN1 applied lime and chemical disinfectant daily following its respondent-reported 2025 ASF episode and used spraying and insecticides to control rodents, birds, and insects. At VN2, disinfection frequency increased from once a week to three times a week during the nationwide ASF outbreak in 2025, while rodent and bird control was conducted quarterly. VN3 used lime and spray disinfectant approximately twice a month. The commercial and integrated units combined manual procedures with specialised facilities. VN4 used automated vehicle cleaning and disinfection equipment and drying rooms for incoming materials. VN6 reported daily disinfection following its respondent-reported 2024 ASF episode and included vehicles, materials, and other production links in its cleaning and disinfection procedures. VN5 required vehicles entering the farm to be cleaned and disinfected.

VN7 and VN8 used rodent-proof walls and protective netting around their production areas. At the time of the visits, damaged sections of netting allowed potential entry by birds or rodents. Vehicle disinfection at these units was applied mainly to the tyres; the interview and observation records indicated that the chassis and some equipment were not included in the same procedure.

#### 3.3.3. Vehicle, Material, Feed, and Pig-Movement Practices

At VN2, feed-delivery and pig-transport vehicles were not kept separate, and traders entered the farm to collect pigs for sale. At VN3, vehicle routes crossed those used by workers. VN4 used designated vehicles and separate loading areas. VN5 reported handover points outside the farm for breeding pigs and fattening pigs, located more than two kilometres and approximately 500 m away, respectively. Vehicles entering VN5 were cleaned and disinfected. VN6 included vehicles and incoming materials in its daily cleaning and disinfection procedures. At VN7 and VN8, vehicle disinfection focused mainly on tyres, with the chassis and some associated equipment left untreated. VN2 received bagged feed and had no feed silo. VN3 reused feed bags. These records provided details on feed handling, but information on feed sources and swill use remained limited across the Vietnamese units. Pig sourcing and restocking procedures were also incompletely documented.

#### 3.3.4. Vaccination, Sick-Pig Isolation, and Carcass Disposal

Two of the eight Vietnamese units reported ASF vaccination; none of the three family farms did so. The units continued to use routine vaccines against other diseases, including porcine reproductive and respiratory syndrome, foot-and-mouth disease, and respiratory infections.

Among commercial and integrated units, VN4 did not report ASF vaccination. VN5 reported that a programme it described as government supported provided the first 100 ASF vaccinations free of charge, generally at two to three months of age. The respondent said 20 of those pigs subsequently died and that mortality continued among piglets of the same age. The programme name, funding mechanism, vaccine manufacturer, vaccine records, and causes of death could not be independently verified; these accounts do not establish a vaccine adverse event. VN6 reported vaccination of sows, piglets, and fattening pigs with products said to be manufactured mainly in Vietnam and also reported another ASF-like episode after restocking. Comparable vaccination information was not available for VN7 and VN8.

Respondents also described divergent responses to suspected sick or dead pigs. VN1 and VN5 reported isolating animals showing clinical signs. One family-unit respondent described selling sick or suspected ASF-affected pigs at a reduced price; another described burial on the premises followed by restocking and further illness. VN5 reported burying pigs believed to have died from ASF with lime, using cooked meat from pigs that survived illness as fish feed, and selling pigs that stopped eating for three consecutive days at a discount. These are unverified respondent accounts. If pigs were infected, informal sale, slaughter, or burial could expose other holdings and undermine notification and safe disposal [11,37].

All Vietnamese units reported strengthening at least one preventive practice after ASF. Structural investment was concentrated among integrated enterprises. However, the sampled cases highlighted continuing challenges related to residual risks, which included trader and vehicle entry, crossed personnel and vehicle routes, incomplete whole-vehicle disinfection, damaged exclusion netting, informal sale or burial of affected pigs, and uncertainty among some producers regarding ASF vaccination outcomes.

### 3.4. Farm-Level Biosecurity Measures in Malaysia

#### 3.4.1. Farm Housing, Zoning, and Personnel Access

Among MY6–MY9, three were commercial farms and one was family-operated. The family-operated unit was managed by household members together with employed foreign workers. The available field records did not document a formal personnel-entry or zoning procedure for this unit. Among the commercial units, one converted its production facilities from a semi-closed system to a fully closed system. The other commercial units also reported modifications to farm infrastructure, although comparable information on internal zoning, worker accommodation, and entry procedures was not recorded for every unit.

Among MY3–MY5, the two family-operated units reported using closed management and prohibiting outside visitors from entering the production area. One of these units invested approximately MYR 2 million in closed house upgrading after experiencing ASF. The commercial unit among MY3–MY5 undertook a broader farm upgrade following reported culling in 2023, with reported expenditure of approximately MYR 6–7 million. It also reduced the number of workers and increased the frequency of staff training.

MY1 and MY2 did not allow outside visitors to enter the premises. Employees were required to remain in isolation for three days before entering the farms and subsequently shower and change into designated work clothing before entering the production areas. One of these respondents operated five farm sites and reported closed-house upgrading at each site, with expenditure of approximately MYR 30,000–50,000 per site. Both producers also reported reducing visits to other farms and direct contact with other pig producers after ASF occurred in Malaysia.

MY10–MY12 had been completely depopulated at approximately the time of the 2022 outbreaks and were not operating during fieldwork. No current housing, zoning, or personnel-entry procedures could therefore be observed. The producers stated that, if restocking were authorised, they intended to construct closed pig houses and operate under updated farm biosecurity procedures.

#### 3.4.2. Cleaning, Disinfection, Pest Control, and Environmental Management

All nine operating Malaysian units reported routine or intensified cleaning and disinfection during the study period. The family-operated unit among MY6–MY9 increased the frequency of whole-farm disinfection after experiencing ASF in 2022. It also increased the observation of pigs for clinical signs. The commercial units among MY6–MY9 reported several investments in cleaning and disinfection infrastructure. One unit spent approximately MYR 360,000 on disinfection equipment and approximately MYR 700,000 on the construction of a cleaning and disinfection centre as part of a larger farm upgrade. Another unit invested approximately MYR 200,000 in upgrading a disinfection pool. The same unit constructed a wastewater separation pond at a reported cost of approximately MYR 1 million. The two family-operated units among MY3–MY5 reported increasing the frequency of disinfection following their ASF experience. At the commercial unit, monthly expenditure on disinfection increased from approximately MYR 15,000 before the outbreak-related changes to approximately MYR 30,000 during the period covered by the interview. MY1 and MY2 reported carrying out disinfection daily. One of these units also installed vehicle-drying equipment, which was used after vehicle cleaning and disinfection. MY10–MY12 had no ongoing cleaning or disinfection programme at the time of fieldwork because the premises remained empty. Their stated plans for restocking included upgrading existing disinfection systems.

Comparable information on rodent, bird, and insect control was not systematically recorded for the Malaysian units. Wastewater-management information was available for one of MY6–MY9, which had constructed a separation pond, but standardised information on manure storage, effluent treatment, and drainage was not available for all operating units.

#### 3.4.3. Vehicle, Material, Feed, and Pig-Movement Practices

Several units reported measures intended to reduce the entry of external livestock vehicles. The family-operated unit among MY6–MY9 purchased two vehicles for its own use after experiencing ASF. These vehicles were used in place of external transport providers for farm-related movements. MY1 and MY2 moved pigs to a handover point outside the farm boundary when pigs were sold. External transport vehicles therefore did not enter the main farm area. One of these units also installed vehicle-drying equipment at a reported cost of approximately MYR 700,000. Vehicles were cleaned and disinfected before the drying procedure. One respondent among MY3–MY5 reported that a slaughterhouse was located within the local pig-production area, less than two kilometres from several farms. According to the respondent, slaughterhouse workers moved between the slaughterhouse and surrounding premises without following a consistent disinfection procedure. The two family-operated units among MY3–MY5 also described changes in feed-related practices. One unit added commercially marketed immune-support products to pig feed. Another producer reported using Chinese herbal products and fermented sour soup as feed additives. These measures are reported as producer-described husbandry practices. MY10–MY12 had no active transport, feed-delivery, or pig-marketing movements during fieldwork because they had not resumed production.

#### 3.4.4. Vaccination, Sick-Pig Isolation, and Carcass Disposal

ASF vaccines were not commercially available in Malaysia during fieldwork, and none of the 12 interviewed producers reported using an ASF vaccine. A family-operated farm among MY6–MY9 reported an illness episode in 2022 in which 10 pigs died. The producer subsequently monitored pigs more closely for clinical signs and reported burying the carcasses away from the main farm area. For the two family-operated farms among MY3–MY5, the study materials contained no official record of an ASF outbreak. Both respondents nevertheless described illness that they believed was ASF and reported that they had not notified the authorities at the time. One lost approximately half the herd, buried carcasses on the farm, and later sold the surviving pigs for approximately MYR 200 per head. Laboratory records were unavailable to confirm these reports of suspected ASF.

The commercial farm among MY3–MY5 reported the culling of 9336 pigs in 2023. Neither MY1 nor MY2 reported ASF infection on their farms. Procedures for isolating sick pigs or disposing of carcasses were not documented for these two units. MY10–MY12 had been fully depopulated following ASF. Operating Malaysian farms reported investment in closed housing, farm-owned vehicles, dedicated disinfection centres, pressure-washing equipment, and wastewater facilities. One producer reported an increase in monthly disinfection expenditure from approximately MYR 15,000 to MYR 30,000. Remaining concerns included financial incentives to avoid reporting, concentrated farm and slaughterhouse traffic, possible exposure to wild boar, and uncertainty about restocking requirements and approval times.

### 3.5. Farm-Level Biosecurity Measures in the Philippines

#### 3.5.1. Farm Housing, Zoning, and Personnel Access

PH1 followed a farrow-to-finish production model. Following reported ASF outbreaks in 2020 and 2023, the farm introduced closed access management and restrictions on personnel entry and exit. PH2 kept three pigs in separate housing and did not use swill feed. PH3 kept 13 pigs, although the interview records did not provide comparable details on the design of its pig housing or personnel-entry arrangements. PH4 operated a small farrow-to-finish system and kept nine pigs, comprising four sows, one boar, and four fattening pigs. Following participation in community-based ASF training, the producer used dedicated slippers and work clothing when entering the pig-rearing area. The reported entry procedure included handwashing, disinfection, and showering before contact with the pigs. Work clothing used within the production area was disinfected once every three days.

#### 3.5.2. Cleaning, Disinfection, Pest Control, and Environmental Management

PH1 reported increasing cleaning and disinfection after its ASF outbreaks. At fieldwork, vehicles, incoming materials, and the premises were disinfected twice daily at a reported cost of approximately PHP 10 per pig per application, around three times the pre-outbreak level. When ASF was reported nearby, PH1 also applied lime and conducted additional environmental disinfection. PH4 likewise reported more frequent disinfection after a suspected ASF event. Its disinfectant cost approximately PHP 720 per litre, and one container lasted about four months; the first supply was provided through the CABI-supported programme, whereas later supplies were purchased by the producer. PH4 also reported handwashing, disinfection, and showering before entry and disinfecting dedicated work clothing every three days. Comparable information on routine disinfection, pest control, manure management, and wastewater treatment was not recorded for PH2 or PH3.

#### 3.5.3. Vehicle, Material, Feed, and Pig-Movement Practices

PH1 owned its livestock-transport vehicles and arranged the transport and sale of pigs without relying on external livestock vehicles. Vehicles and incoming materials were included in the twice-daily disinfection procedure. Feed practices were recorded for PH2 and PH3. PH2 did not use swill feed and kept its pigs in separate housing. Both PH2 and PH3 used locally grown, high-protein forage grass as a feed source. PH4 sold pigs either through traders or by direct sale. Motorcycles were normally used for direct sales. Under normal production conditions, pigs were sold at approximately PHP 160 per kg and at an average live weight of about 70 kg.

#### 3.5.4. Vaccination, Sick-Pig Isolation, and Carcass Disposal

Vaccination and disease histories were based on respondent reports. All four Philippine units reported routine vaccination against other pig diseases, including mycoplasmal pneumonia and porcine reproductive and respiratory syndrome (PRRS). PH1 also reported using an ASF vaccine supplied through a government-supported programme, with vaccination schedules varying by pig category. PH2 and PH3 did not report ASF episodes or provide comparable details of ASF vaccination schedules. PH4 described a suspected ASF episode involving four sick pigs. The respondent reported that the pigs were privately slaughtered and the pork distributed to neighbours. The remaining apparently healthy pigs were sold for approximately PHP 150 per kg, below the reported market price of about PHP 170 per kg. No laboratory results, formal notification records, or records of authorised disposal were available for this episode.

PH1 reported increased spending on routine prevention, including twice-daily disinfection, farm-owned transport, entry restrictions, and vaccination. The backyard units used lower-cost household measures. PH2–PH4 also received training and a limited supply of materials through the CABI-supported programme. Remaining concerns included dispersed backyard production, movements of traders and motorcycles, limited knowledge of outbreak response, and private slaughter and pork distribution following suspected disease. Respondents also expressed low confidence in compensation and formal reporting arrangements.

### 3.6. Cross-Country Recovery, Investment, and Residual Risks

Table 4 brings together recovery patterns, reported post-ASF investment, residual risks, and public or project support in the sampled cases. The evidence comes mainly from interviews and field notes; national statistics appear only as explicitly labelled context. Cleaning and disinfection were the most consistently documented measures. Pig movement, carcass and environmental management, reporting, compensation, and restocking were documented less consistently.

Respondents in all three countries described increased biosecurity inputs, but monetary evidence was uneven. Before-and-after expenditure was clearest for Malaysian operating farms and PH1. Vietnamese integrated enterprises and Malaysian operating farms reported major structural changes, whereas Vietnamese family farms and Philippine backyard units more often described recurrent, lower-cost measures. Reported external support comprised a government-supported vaccination arrangement at VN5 whose details could not be independently verified, vaccination at PH1, and CABI-linked training and materials for PH2–PH4. Malaysian respondents described public intervention chiefly through regulation, inspection, and restocking approval rather than a documented capital grant.

**Table 4:** **Cross-country comparison of recovery, post-ASF investment,** **residual risks, and support observed in the sampled cases.**

Country	National Context and Case Findings	Farm-Level Investment After ASF	Main Residual Risks	Reported Public or Project Support
Vietnam	National context: output exceeded the 2018 level, while inventory remained lower [24]. Case findings: VN5 and VN6 remained below their reported pre-outbreak herd sizes.	Integrated enterprises added zoning, controlled loading, material drying, and automated vehicle disinfection. Family farms mainly increased lime and chemical disinfection (VND 120,000–1 million per application).	Traders and shared vehicles; crossed routes; partial vehicle disinfection; informal sale/burial; variable confidence in vaccine performance.	VN5 reported free vaccination for the first 100 pigs; national veterinary control and vaccination policy continued.
Malaysia	National context: inventory and pork output remained below 2020 levels [26]. Case findings: nine farms were operating; MY10–MY12 remained depopulated awaiting restocking approval.	Closed-house and site upgrades (MYR 30,000–50,000 per site; MYR 2 million and MYR 6–7 million in two cases), farm-owned vehicles, disinfection centres, and wastewater facilities. One farm’s monthly disinfection cost rose from MYR 15,000 to MYR 30,000.	Perceived disincentives to reporting were described by some respondents; clustered farm/slaughter traffic; possible wild-boar contact; uncertain timing and criteria for restocking.	Disease-control rules, inspection, and restocking approval were reported; no direct public investment grant was identified in the sampled cases.
Philippines	National context: inventory was 8.79 million head at the end of 2025 versus 12.80 million at the start of 2020 [8,25]. Case findings: all four units were operating; comparable pre-outbreak herd sizes were unavailable.	PH1 increased disinfection to twice daily, used farm-owned vehicles, and reported vaccination. Backyard units mainly purchased disinfectant or used other low-cost household measures.	Traders and motorcycles; feed-source information gaps; private slaughter and pork distribution; compensation and reporting concerns. Wider context: dispersed production and inter-island movement.	PH1 reported government-supported vaccine access; PH2–PH4 received CABI-supported training and a limited materials package.

Notes: Case findings derive from respondent accounts and field notes; national inventory/output figures are contextual official statistics and are not case outcomes. Monetary values are respondent-reported in local currency.

### 3.7. Risk Appraisal, Coping Capacity, and Reporting Behaviour

Most respondents recognised that ASF could cause high mortality, compulsory culling, business interruption, and delayed restocking. However, awareness of severity did not consistently coincide with perceived susceptibility, confidence in available measures, capacity to act, or structural investment. Interpreted through PMT, outbreak experience appeared to strengthen threat appraisal without necessarily making effective responses affordable or operationally feasible.

Risk appraisal and coping capacity differed across cases. Five of the eight Vietnamese units—mainly integrated enterprises—described ASF as a high risk, whereas VN1–VN3 did not clearly characterise their own farms as facing an immediate high risk. One previously affected producer reduced contact with other pig farmers but relied heavily on prior experience and did not consistently change footwear or clothing before entry. All 12 Malaysian respondents regarded ASF as a high-risk event, citing infection as well as the consequences of detection, including whole-herd culling, closure, and delayed re-entry. In the Philippines, PH1 expressed high concern and used a coordinated set of controls; PH2–PH4 expressed less concern and had more limited knowledge of transmission and outbreak response. Viewed through PMT, perceived severity was widespread, but response efficacy and self-efficacy appeared more uneven. Integrated enterprises, operating Malaysian farms, and PH1 described greater capacity to implement housing, personnel, vehicle, and material controls, whereas Vietnamese family farms and Philippine backyard units relied more on experience and lower-cost practices. These observations are qualitative interpretations, not direct measurements of PMT constructs.

Reporting accounts were also compatible with TPB and incentive constraints. Penang respondents associated notification with whole-herd culling, prolonged closure, and possible investigation of neighbouring farms, and some described non-reporting in the production area. Vietnamese and Philippine respondents described low-price sale, private slaughter, burial, or other handling outside formal response channels. Producers described limited control over culling scope, payment timing, and restocking conditions. Seventeen of the 24 units reported some understanding of ASF and its main transmission pathways, usually from prior outbreaks, online sources, or other producers. Formal government- or project-linked training was reported by PH2–PH4 and VN6, a government-linked Vietnamese enterprise. Other units did not report comparable training, although officials described local veterinary authorities as responsible for technical guidance. These are respondent reports, not measured knowledge scores.

## 4. Discussion

Recovery remained uneven among the sampled units despite reported increases in biosecurity investment. Vietnamese integrated enterprises introduced structural controls, although some continued to operate below their earlier herd sizes. Several Malaysian farms upgraded their facilities, while three remained depopulated awaiting restocking approval. Philippine backyard units maintained production with household measures and project support. Farm scale and resources are relevant to production recovery and disease control [38,39]. Integrated enterprises and several commercial farms invested in controlled entry, dedicated transport, and specialised disinfection facilities while family and backyard units more often relied on measures requiring limited initial expenditure. Research in Myanmar has documented financial and veterinary-service constraints on household disease control [17]. A review of ASF prevention in smallholder pig systems identified similar barriers to biosecurity [18]. Milton et al. documented recurrent ASF outbreaks and estimated associated economic losses in Meghalaya, India, where backyard production is common [40]. Repeated losses may further restrict the resources available for prevention and restocking. In the Philippine cases, CABI provided training and materials, although continued implementation also required household spending and daily labour.

Risk awareness and the ability to act were closely connected in the interview accounts. Under PMT, confidence in the effectiveness and feasibility of a response helps explain why producers facing similar disease threats adopted different measures [19,20]. Reporting decisions involved additional costs. Respondents described concerns about compulsory culling, business interruption, compensation, and uncertain restocking dates. These concerns are relevant to perceived control under TPB [21] and to the distribution of disease-control costs and benefits [22,23]. A Philippine policy analysis discusses the financial and practical difficulties of ASF stamping-out [41]. Research with Malaysian pig farmers found that economic, social, and institutional factors shaped disease-control decisions [42]. For MY10–MY12, uncertainty over approval remained a practical obstacle to planning further investment.

The emphasis on disinfection in many interviews needs to be considered alongside the coverage of cleaning procedures. At VN7 and VN8, vehicle treatment focused mainly on tyres, leaving potential contamination on other surfaces insufficiently addressed. ASFV can remain infectious in blood-derived material, supporting careful removal of contamination from vehicles, footwear, tools, and equipment [43]. In laboratory suspension tests, 1% sodium hypochlorite reduced infectious ASFV titres by more than 4 log_10_ after 30 min at 10 °C under both low- and high-soiling conditions [44]. Field procedures require prior cleaning and a disinfectant concentration and contact time validated for the intended surfaces and conditions. Other documented practices concerned direct contact and oral exposure. Trader entry, shared transport, and crossings between personnel and vehicle routes create opportunities for transferring contaminated material. Known-source pigs, pre-entry isolation, controlled loading, and separation of transport routes can reduce these contacts [11,37]. Private slaughter and distribution of pork from suspected pigs, as reported at PH4, may extend exposure beyond the affected household. Prompt reporting, safe carcass collection, and authorised disposal are therefore important alongside farm entry controls.

Wildlife contact also warrants attention where farms border wild-pig habitat. In Europe, infected wild boar, carcasses, and contaminated sites can sustain transmission and amplify environmental exposure [11,37]. Investigations in Lao PDR and Vietnam detected ASFV in wild-boar carcasses near areas where domestic pigs overlapped with wild-boar habitat [45]. The extent of sustained wildlife transmission in Southeast Asia remains uncertain. Farms in these settings need barriers that prevent contact with wild pigs, secure storage of feed and waste, and carcass disposal that limits wildlife access. Reporting and testing dead wild pigs would help clarify local transmission patterns.

ASF vaccination requires continued attention to circulating virus strains. Recombinant genotype I/II ASFV was detected in domestic pigs in Vietnam in 2023 [46]. A Vietnamese isolate with genetic features consistent with an ASFV-G-ΔI177L vaccine origin has been reported, suggesting possible evolution of vaccine-derived virus under field conditions [47]. Co-infection with vaccine and field viruses raises concern about recombination and possible changes in virulence or immune protection [48]. Establishing these outcomes requires genomic and experimental evidence. Challenge experiments have already shown incomplete protection by two genotype II vaccine strains against a recombinant genotype I/II virus [49]. These findings support recording vaccine products, batches, doses, and dates, together with investigation of illness or deaths following vaccination. WOAH recommends post-vaccination monitoring and genomic surveillance to detect possible reversion or recombination [48].

Practical support should address the difficulties identified in each setting. For the Vietnamese cases, priorities include affordable loading arrangements, vehicle cleaning, and access to safe disposal. Malaysian producers awaiting restocking would benefit from clear inspection requirements, approval stages, and decision timelines. For Philippine backyard producers, community training should include follow-up visits, accessible reporting channels, and assistance with suspected cases. Respondents’ concerns about the financial consequences of notification support clear compensation procedures and information on eligibility. A Philippine policy analysis discusses difficulties with stamping-out and compensation [41]. A study of US swine producers also examined how indemnification relates to attitudes towards ASF prevention [50].

## 5. Conclusions

This study found uneven recovery among the selected production units. In Vietnam, national pig output exceeded its 2018 level while inventory remained lower; within the sample, integrated enterprises invested more heavily than family farms, yet restoration of reported herd size remained uneven. Malaysian operating farms described substantial structural upgrades, but three depopulated units were still awaiting permission to restock. In the Philippines, the commercial case reported coordinated controls, whereas three programme-linked backyard households relied on smaller-scale measures and external support. The policy review identified different national emphases: disease-free production chains and vaccination in Vietnam, controlled restocking and housing upgrades in Malaysia, and barangay-level surveillance and community support in the Philippines. The cases cannot establish which framework was most effective. The cases highlight the importance of feasible farm practices and access to notification, authorised disposal, movement control, and restocking procedures. Because disease histories and many biosecurity practices were self-reported and incompletely documented, the findings apply to the sampled units and cannot establish national prevalence or causal policy effects.

## Figures and Tables

**Table 1:** **Characteristics of the** **sampled pig-production units.**

Production Unit	Location	Production System	Reported Herd Size or Composition	Status at Fieldwork
VN1	Thanh Hoa	Family farm	100 sows; 700 fattening pigs (800 total)	Operating
VN2	Thanh Hoa	Family farm; farrow-to-finish	100 sows; 600 fattening pigs (700 total)	Operating
VN3	Hung Yen	Family finishing farm	200 fattening pigs	Operating
VN4	Hung Yen	Integrated enterprise	Current standing herd NR	Operating
VN5	Ho Chi Minh	Commercial farrow-to-finish enterprise	Current: ~200 sows and ~2000 fattening pigs (~2200 total); pre-ASF: nearly 4000	Operating after restocking
VN6	Ho Chi Minh	Multi-site commercial enterprise	Current: <7000 sows and ~23,000 fattening pigs; pre-outbreak: ~100,000	Operating after restocking
VN7	Hai Duong	Integrated enterprise	NR	Operating
VN8	Hai Duong	Integrated enterprise	NR	Operating
MY1	Selangor	Pig farm	10,000 pigs, including 1000 sows	Operating
MY2	Selangor	Pig farm	2400 pigs, including 240 sows	Operating
MY3	Penang	Pig farm	10,000 pigs, including 1000 sows	Operating
MY4	Penang	Pig farm	1000 pigs, including 100 sows	Operating
MY5	Penang	Pig farm	2000 pigs, including 200 sows	Operating
MY6	Perak	Pig farm	10,000 pigs, including 800 sows	Operating
MY7	Perak	Pig farm	20,000 pigs, including 1800 sows	Operating
MY8	Perak	Pig farm	40,000 pigs, including 3500 sows	Operating
MY9	Perak	Pig farm	2100 pigs, including 240 sows	Operating
MY10	Melaka	Depopulated pig farm	No pigs at fieldwork; pre-depopulation herd NR	Not restocked
MY11	Melaka	Depopulated pig farm	No pigs at fieldwork; pre-depopulation herd NR	Not restocked
MY12	Melaka	Depopulated pig farm	No pigs at fieldwork; pre-depopulation herd NR	Not restocked
PH1	Luzon	Large commercial, family-operated; farrow-to-finish	1500 breeding sows; total standing herd NR	Operating
PH2	Dumaguete area, (community withheld)	Community-based backyard unit	3 pigs	Operating
PH3	Dumaguete area, (community withheld)	Community-based backyard unit	13 pigs	Operating
PH4	Dumaguete area, (community withheld)	Backyard farrow-to-finish unit	9 pigs: 4 sows, 1 boar, and 4 fattening pigs	Operating

Notes: NR, not reported. Herd size refers to standing inventory at fieldwork unless otherwise indicated. Current herd size was not recorded for VN4, VN7, or VN8 (3/8 Vietnamese units). VN6 figures refer to combined inventory across three sites. MY10–MY12 had no pigs at fieldwork following depopulation; their production-system subtype was not recorded.

## Data Availability

The data presented in this study are available on request from the corresponding author, subject to participant confidentiality, ethics requirements, and any restrictions imposed by the organisations involved.

## Supplementary Materials

The following supporting information can be downloaded at: https://www.mdpi.com/article/10.3390/vetsci13101113/s1, Supplementary File S1. African Swine Fever (ASF) Impact Survey on Pig Farms.
