# Supplementary material for: Implementation of African Swine Fever Biosecurity and Post-Outbreak Recovery in Vietnam, Malaysia, and the Philippines
Source: Vet Sci. 2026 Oct 18;13(10):1113. doi: 10.3390/vetsci13101113 (PMC13709924; doi:10.3390/vetsci13101113)
Supplement: Supplementary file 1 [file vetsci-13-01113-s001.zip › vetsci-4587584-supplementary.pdf]

Supplementary File S1. African Swine Fever (ASF) Impact Survey on Pig Farms

Country: _______; (Province/City): _________ ; Year of Farm Establishment: _______ ; Type of Farming Entity: _______ (Family Farm, Enterprise); Farming Type: _______ (Farrow-to-Finish, Fattening, Mixed); Farming Model: _______ (Independent Farmer, Contract Farmer, Market-Oriented Farmer [combination of contract and self-sales])

Topographical Features: _______

I. Basic Information

Q1. What is the current pig inventory (including sows, piglets, and fattening pigs) on your farm? (Fill in) _______ heads; among them, how many sows? _______ heads.

Q2. What is the production cost for pigs marketed in the first half of 2025? (Fill in) _______ (per head/kg, in local currency/USD); Live weight at slaughter: _______ kg. Which year in the past few years had the highest production cost? _______; Cost amount: _______ (per head/kg).

Q3. What is the current live pig price per 500g (jin)? (Fill in) _______ (local currency/USD).

Q4. What is the sales channel for marketing pigs? (Fill in) ________________ (Self-marketing / Dealer-organized transportation / Cooperative / Contract enterprise).

Q5. When selling pigs, are they loaded onto the truck inside the farm or driven outside the farm before loading?

Q6. How do you dispose of sick or dead pigs?

Q7. If there is an ASF outbreak in surrounding farms, how would you handle the pigs currently in your barns?

II. Biosecurity and Control Measures

Q8. Has your farm established a standard biosecurity Standard Operating Procedure (SOP)?

Q9. Do you implement closed management (access control, visitor management)? (Single choice) ○ Yes ○ No

Q10. Main preventive measures (Multiple choices):
□ Strict control of personnel entry and exit
□ Isolation between production and living areas
□ Increased frequency of disinfection for vehicles and materials
□ Enhanced disinfection frequency during vacancy and daily operations

□ Quarantine of introduced breeding stock
□ Feed inspection and disinfection
□ Harmless disposal of dead pigs
□ Training on epidemic prevention
□ Others: ________

Q11. Do pigs on the farm receive ASF vaccination? If so, does the government provide subsidies? What is the cost? What is the effectiveness after vaccination?

Q12. How often do you disinfect the farm? What is the cost? Compared with 2018 (or before ASF), has the frequency and cost of disinfection increased? If so, by how much?

III. Biosecurity Upgrades

Q13. Has your farm implemented structural or technical upgrades to prevent ASF? If yes, please select the items upgraded (Multiple choices):
□ Farm layout modification (separation of clean/dirty routes, isolation barns)
□ Addition of automated farming equipment
□ Purchase of disinfection equipment
□ Road hardening
□ Reinforcement for rodent and bird control
□ Others: ________________________________

Q14. Total investment in biosecurity upgrades: ____________________________ (in 10,000 local currency units).

IV. Economic Impact

Q15. Changes in your farm in the following aspects (unit: 10,000 local currency units/year):

Item	Investment Amount	Remarks
Facility and infrastructure upgrades		
Disinfection		
Vaccination and testing		
Staff training and management		
Epidemic prevention materials		
Others (please specify)		

Q16. Economic impact: What has been your farm's profitability in recent years? Profit or loss? Which year had losses? Which year had profits? Will you continue pig farming?

V. Policies and Support

Q17. Are you familiar with national ASF prevention and control policies? (Single choice)
○ Very familiar
○ Somewhat familiar
○ Not familiar

Q18. Have you received any of the following support? (Multiple choices):
□ Government subsidies
□ Technical guidance
□ Equipment support
□ Training programs
□ Others: ________

Q19. If you received subsidies, what type were they? (Cull compensation, insurance subsidies, loan subsidies, land use subsidies, etc.)
